# Adenoviral-vectored epigraph vaccine elicits robust, durable, and protective immunity against H3 influenza A virus in swine

**DOI:** 10.3389/fimmu.2023.1143451

**Published:** 2023-05-15

**Authors:** Erika Petro-Turnquist, Matthew Pekarek, Nicholas Jeanjaquet, Cedric Wooledge, David Steffen, Hiep Vu, Eric A. Weaver

**Affiliations:** ^1^ Nebraska Center for Virology, University of Nebraska-Lincoln, Lincoln, NE, United States; ^2^ School of Biological Sciences, University of Nebraska-Lincoln, Lincoln, NE, United States; ^3^ Office of Research and Development, University of Nebraska-Lincoln, Lincoln, NE, United States; ^4^ Nebraska Veterinary Diagnostic Center, Lincoln, NE, United States; ^5^ Department of Animal Science, University of Nebraska-Lincoln, Lincoln, NE, United States

**Keywords:** adenoviral (Ad) vector, epigraph, swine influenza, vaccine, duration, transmission, pathology

## Abstract

Current methods of vaccination against swine Influenza A Virus (IAV-S) in pigs are infrequently updated, induce strain-specific responses, and have a limited duration of protection. Here, we characterize the onset and duration of adaptive immune responses after vaccination with an adenoviral-vectored Epigraph vaccine. In this longitudinal study we observed robust and durable antibody responses that remained above protective titers six months after vaccination. We further identified stable levels of antigen-specific T cell responses that remained detectable in the absence of antigen stimulation. Antibody isotyping revealed robust class switching from IgM to IgG induced by Epigraph vaccination, while the commercial comparator vaccine failed to induce strong antibody class switching. Swine were challenged six months after initial vaccination, and Epigraph-vaccinated animals demonstrated significant protection from microscopic lesion development in the trachea and lungs, reduced duration of viral shedding, lower presence of infectious virus and viral antigens in the lungs, and significant recall of antigen-specific T cell responses following challenge. The results obtained from this study are useful in determining the kinetics of adaptive immune responses after vaccination with adjuvanted whole inactivated virus vaccines compared to adenoviral vectored vaccines and contribute to the continued efforts of creating a universal IAV-S vaccine.

## 1 Introduction

Swine Influenza A Virus (IAV-S) is a significant pathogen that affects swine populations around the world (1) and imposes a significant burden on the pork industry. Economic losses to the U.S. pork industry can cost nearly $700,000,000 per year (2), and are due to reduced weight gain, delayed production, and increased susceptibility to secondary infections leading to greater veterinary costs (3–5). At present IAV-S in swine is considered one of the three top health challenges to the swine industry (6) and affects swine in all phases of production (7). Three main subtypes of IAV-S currently circulate in U.S. swine populations: H1N1, H1N2, and H3N2 (8). Though a large proportion of circulating strains are of the H1N1 subtype, recent epidemiological surveys indicate an increased incidence of H3N2 IAV-S detected in U.S. swine (9). IAV-S causes acute respiratory disease that typically resolves 3-7 days after infection (10) but can cause up to 100% morbidity in infected herds. Additionally, swine are susceptible to swine, avian, and human influenzas due to the distribution of α2, 3- and α2, 6-linked sialic acid receptors in the respiratory tract (11, 12). Because of this, swine are considered “mixing vessels” and can foster reassortment of influenza during co-infections with multiple strains, resulting in the evolution of antigenically distinct and potentially pandemic strains of IAV (3, 13). Interspecies transmission of IAV between swine and humans has been described to occur at slaughterhouses, swine production barns, live animal markets, and even agricultural fairs (14, 15). Zoonotic emergence of IAV was recently named a top priority of the One Health workshop for disease prevention in the United States. More specifically, swine were considered a significant intermediate reservoir in IAV infections and pose the greatest risk of zoonotic transmission of IAV into humans (6). One such example occurred in 2009 and was termed the “swine flu” pandemic. This novel swine influenza isolate initially arose in Mesoamerica, but quickly spread and infected ~24% of the global population within the first year (16). Importantly, this zoonotic transmission event paved the way for the establishment of a new and stable lineage of H1 influenza in humans, now known as H1N1pdm09, and is the most predominantly circulating lineage of H1N1 in humans today (17, 18). Due to the significant role swine play in the evolution and transmission of potentially pandemic strains of influenza and the substantial economic impacts of IAV-S, it is imperative that efforts be made towards the development of more effective vaccination strategies in vulnerable pig populations.

Current methods of controlling IAV-S in swine include commercially available whole-inactivated virus (WIV) vaccines, autogenous herd-specific virus vaccines, and live attenuated influenza virus (LAIV) vaccines. Commercially available WIV vaccines, such as FluSure XP, incorporate 2 strains of H1 and 2 strains of H3 IAV-S and are often supplemented with an oil-in-water adjuvant. While WIV vaccines have shown to induce robust protection after homologous challenge (19–22), interface with antigenically divergent IAV-S can result in dampened cross protection (20). Further, this method of vaccination has been linked with the induction of vaccine-associated enhanced respiratory disease (VAERD) after heterologous challenge (20, 23). This phenomenon is characterized by the presence of cross-reactive, but non-neutralizing antibodies directed towards the HA2 stalk domain of a hemagglutinin protein (24). In the absence of neutralizing antibodies against the HA1 head domain, these antibodies have been described to facilitate enhanced viral infection of MDCK cells *in vitro* and rapidly induce dysregulated levels of proinflammatory cytokines in the lungs (25). Further recruitment of inflammatory cell populations results in collateral damage in the lungs and enhanced respiratory disease. Because the process of producing and licensing a WIV vaccine is time-consuming and expensive, the commercial WIV is not updated fast enough to cope with the continually evolving swine influenza virus. In light of these challenges, autogenous herd-specific WIV vaccines are gaining popularity, with a staggering estimate of 50% of IAV-S vaccines employed in the United States being autogenous WIV vaccines in 2008 (26, 27). However, autogenous WIV vaccines have multiple drawbacks, including labor intensive laboratory techniques for diagnosis, isolation, virus growth, purification, and efficacy testing. This leads to a significant lag period before administration of the vaccine to a given herd. LAIV vaccines have recently been approved for clinical application in swine and promisingly induce heterologous protection where WIV have failed (28, 29). However, evidence of reassortment between the LAIV strain and field IAV-S calls to question the safety of the LAIV platform (30). The inherent difficulties of producing a seasonal vaccine for swine demonstrates that a safe and universal swine influenza vaccine that induces durable and broadly cross-reactive immunity to all divergent strains is needed.

Adenoviruses (Ad) are present in several mammalian species including cows, sheep, pigs, chimpanzees, and humans, and have the ability to naturally infect and replicate in a broad spectrum of cells (31, 32). Infection of epithelial cells at mucosal surfaces and dendritic cells can results in efficient antigen presentation and elicit potent immune responses. Ad has a stable double-stranded DNA genome that is maintained as an episome in an infected cell (33), mitigating the risk of insertional mutagenesis (31). Further, Ads can be made replication-defective by deleting the early E1 gene and replacing it with a gene of interest (34). Ads can be further modified to increase packaging capacity of a desired transgene by deletion of the E3 gene (34), which modulates the host immune response during an infection. Both replication-competent and replication-defective Ad vectors have been investigated as potential vaccine candidates and are superior to inactivated virus vaccines by mimicking a natural viral infection. The induction of cytokines and costimulatory molecules provide a potent adjuvant effect *in vivo* and can elicit robust adaptive immune responses to a delivered transgene. Further, amplification of replication-defective Ad amplification is easily scalable by expansion in E1-complementing cell lines in large bioreactors, and rapid ultracentrifugation purification techniques (32). A recent estimate indicates that large-scale production of Ad-vectored vaccines can cost as little as $1.25/dose (35). However, a common concern with using an adenovirus as a viral vector for vaccine development is the presence of preexisting immunity that neutralizes the vector and causes dampened immunity to the delivered transgene (36). Using adenovirus serotypes with low seroprevalence and non-human adenoviral vectors can address this concern when developing vaccines and therapies for humans. Congruently, the same tactic can be used when creating adenoviral-vectored vaccines for non-human mammalian species. Human adenovirus type 5 (Ad5) has been well described in the swine animal model to prevent foot and mouth disease (FMD) (37), porcine respiratory and reproductive syndrome virus (PRRSV) (38), and pseudorabies virus (PrV) (39, 40), and can serve as an attractive viral vector for a universal vaccine against IAV-S.

To address the need for improved vaccination methods in swine, our group has recently characterized the use of a replication-defective adenoviral-vectored Epigraph vaccine against swine H3 influenza A virus in mice and swine (41). The Epigraph platform uses a computational algorithm to determine the frequency of potential T cell epitopes in a target population of sequences and incorporates the highest frequency epitopes into a synthetic immunogen for optimal T cell activation after immunization (42, 43). Indeed, we have previously shown that this platform induces significantly higher cross-reactive antibodies, robust T cell activation, and protection against divergent swine and human H3 influenza challenge in mice compared to a WT immunogen and the commercial comparator WIV vaccine, FluSure XP (41). However, to the best of our knowledge, no studies have performed a longitudinal study analyzing the onset and duration of immune responses elicited after vaccination with an adenoviral-vectored vaccine and compared these results to a WIV vaccine in swine.

Here, we evaluated the kinetics of antibody and T cell response generation after vaccination with an adenoviral-vectored Epigraph vaccine (Ad-swH3-Epi) and compare the responses observed to vaccination with a commonly used WIV vaccine, FluSure XP. We further characterize the differences in antibody class switching after vaccination with these different platforms. Finally, we assessed protection against challenge 6 months after the initial vaccination to evaluate the extent of the protective responses in a clinically relevant model, as the average lifespan of standard market pig in the pork industry is 6-7 months of age. The data observed in this study support the use of Ad-swH3-Epi for robust and durable protection against H3 IAV-S in swine.

## 2 Materials and methods

### 2.1 Ethics statement

All procedures in this study were approved by the Institutional Biosafety Committee at the University of Nebraska-Lincoln (IBC #619). Fifteen 3-week-old cross bred female Yorkshire pigs serologically negative for prior influenza exposure were obtained from Midwest Research Farms and randomly allocated into three immunization groups. All pigs were housed at the University of Nebraska-Lincoln Life Science Annex under animal biosafety level 2 (ABSL2) conditions (IACUC #2167) as per the Association for Assessment and Accreditation of Laboratory Animal Care International (AAALAC) guidelines with access to food and water *ad libitum* and were allowed to acclimate for one week prior to immunization.

### 2.2 Cells and viruses

The following swine influenza A virus was a generous gift from our collaborator: A/swine/Kansas/11-110529/2011 (sw/KS/11) from Dr. Wenjun Ma. The following viruses were obtained from the Biodefense and Emerging Infectious Diseases Repository: A/swine/Ohio/11SW87/2011 (sw/OH/11) [NR-36715] and A/swine Manitoba/00446/2005 (sw/Man/05) [NR-43049]. The following isolates were obtained from the USDA Swine Surveillance Influenza A virus repository: A/swine/Texas/4199-2/1998 (sw/TX/98), A/swine/Colorado/23619/1999 (sw/CO/99), A/swine/Wyoming/A01444562/2013 (sw/WY/13), A/swine/Minnesota/A01432544/2013 (sw/MN/13), A/swine/Indiana/A01202866/2011 (sw/IN/11), and A/swine/Texas/A01785781/2018 (sw/TX/18). All swine influenza viruses were grown in specific pathogen-free embryonated chicken eggs, quantified by hemagglutination assay (HA) and TCID_50_, and quantified virus from the chorioallantoic fluid was stored at -80°C for subsequent assays.

Madin-Darby Canine Kidney-London strain (MDCK-Ln) cells used in TCID_50_ assays were cultured in DMEM supplemented with 5% FBS and 1% penicillin-streptomycin at 37°C, 5% CO2 in a humidified incubator.

### 2.3 Animal immunization and sampling

**Figure 1:**
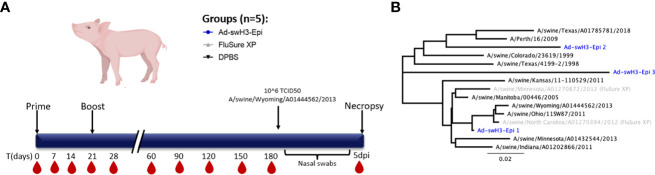
Study timeline and phylogenetic analysis of strains used in study. **(A)** Groups of three-week-old swine (n=5/group) were immunized with Ad-swH3-Epi, FluSure XP, or DPBS as a negative control group at D0 and boosted at D21. Blood samples were collected every 7 days for the first month, then every 30 days for the subsequent 5 months for a total duration study of 6 months. Six months after the initial immunization all pigs were subjected to challenge with a divergent Cluster IV(A) IAV-S isolate, A/swine/Wyoming/A01444562/2013. Nasal swabs were taken every other day after challenge, then at 5 days post infection pigs were humanely bled, euthanized, and samples were taken for analysis. **(B)** Phylogenetic divergence of strains used for serological analysis, T cell analysis, and challenge (black) compared to Epigraph immunogens (blue) and strains incorporated in FluSure XP WIV vaccine (grey).

Production of the Epigraph immunogens has been described previously (41). Briefly, all unique and full-length swine H3 strains as of April 25^th^, 2017 were downloaded from the Influenza Research Database, aligned using ClustalW, and submitted to the Epigraph Vaccine Designer at the Los Alamos National Laboratories with the following parameters: cocktail size: 3, epitope length: 9. On study day 0 (D0), pigs in the Ad-swH3-Epi group were intramuscularly immunized with 10^11^ viral particles (vp) (the cocktail of three epigraph immunogens at equal ratios (3.33x10^10^ vp per epigraph) to a total of 10^11^ vp (41)) diluted in 1 mL DPBS, then boosted three weeks after the prime immunization (D21). Pigs in the FluSure XP group were intramuscularly immunized according to manufacturer’s instructions with 2 mL on D0, then boosted on D21. A group of five pigs served as a negative control group and received a 2 mL intramuscular injection of DPBS on D0 and D21. Whole blood was collected by external jugular vein puncture every 7 days for the first month, every 30 days for the subsequent 5 months, and 5 days after influenza challenge. Serum was isolated from whole blood using BD Vacutainer Separator Tubes (Becton Dickinson) on D0, D7, D14, D21, D28, D60, D90, D120, D150, and D180 post-vaccination (**Figure 1A**). PBMCs were isolated on D0, D21, D60, D120, D150, D180, and 5 days post infection (5dpi) by diluting whole blood 1:1 with sterile DPBS then gently layering diluted blood on top of lymphocyte separation media (Corning #25072CV) and centrifuging diluted blood at 400g for 30 minutes. The PBMC layer was collected, washed with RPMI containing 5% FBS and 1% penicillin streptomycin, and residual red blood cells were lysed with ACK lysis buffer for 5 minutes before quenching with complete RPMI. Cells were resuspended in complete RPMI, counted on a Coulter Counter (Beckman Coulter), then cryopreserved in freezing medium containing 50% RPMI, 45% FBS, and 5% DMSO.

### 2.4 Hemagglutination inhibition assay

Sera from whole blood was used for hemagglutination inhibition (HI) activity according to previously described methods (44). Briefly, sera were treated in a 1:3 ratio with receptor destroying enzyme (RDE; Denka Seiken) at 37°C for 18 hours. RDE was then heat-inactivated at 56°C for 1 hour, and serum was diluted to a final 1:10 dilution in sterile DPBS. Sera were then serially diluted 2-fold in a 96-well V-bottom plate, and 4 hemagglutination units (HA) of representative swine influenza virus from Cluster I, II, IV, IV(A), IV(B), IV(C), IV(F), or the 2010.1 human-like cluster (**Figure 1B**) added to the serum dilutions and incubated at room temperature for 1 hour. 50μL of 0.5% chicken red blood cells was added to each well and hemagglutination patterns were read after 30 minutes.

### 2.5 IgM and IgG antibody isotyping by recombinant protein ELISA

IgM and IgG antibody responses were analyzed against A/Perth/16/2009 HA protein by ELISA. Briefly, flat-bottomed 96-well plates (Immunolon 4 HBX; VWR) were coated overnight at 4°C with 100μL (150ng/well) of recombinant A/Perth/16/2009 HA protein (NR-49734) diluted in carbonate/bicarbonate coating buffer. Wells were washed 4 times with PBS containing 0.1% Tween 20 (PBS-T) then blocked at room temperature with blocking buffer (10% skim milk diluted in PBS-T) for 2 hours. Sera samples were heat-inactivated at 56°C for 1 hour, then serially diluted two-fold in 5% skim milk in PBS-T and 100μL of each dilution was added to the coated wells and incubated at room temperature for 1 hour. Plates were washed 5 times with PBS-T then HRP-conjugated goat anti-pig IgM (Cat. No. AAI48P; BioRad) or IgG (Cat. No. AHP865P; BioRad) antibody diluted to 1:5000 in 5% skim milk in PBS-T were added to wells and incubated at room temperature for 30 minutes. Plates were washed 5 times with PBS-T then developed with 1-Step Ultra TMB-ELISA (Thermo Fisher) and the reaction was stopped with 2M sulfuric acid. Absorbance values were evaluated at OD450 on a SpectraMax i3x Multi-Mode automatic microplate reader (Molecular Devices). Endpoint titers were calculated as equivalent to the mean plus three standard deviations of the OD values from the PBS-immunized control animals at each timepoint.

### 2.6 IFN-γ ELISPOT analysis

PBMCs were analyzed for T cell responses by IFN-γ ELISpot assay. An overlapping peptide array spanning the entire length of A/swine/Ohio/11SW87/2011 HA protein was synthesized by Genscript as individual 17-mer peptides with 10 amino acid overlaps. 96-well polyvinylidene difluoride-backed plates (MultiScreen-IP, Millipore) were coated with anti-porcine IFN-γ mAb pIFN-γ (5μg/mL; Mabtech) at 4°C overnight. Plates were washed three times with DPBS then blocked with RPMI containing 10% FBS and 1% penicillin and streptomycin for 2 hours at 37°C. Single-cell suspensions of 2.5x10^5^ PBMCs 2.5 x 10^5^ cells were stimulated overnight at 37°C, 5% CO2 with 5μg/mL peptide, concanavalin A (ConA; 5μg/mL), or RPMI, in duplicate. After overnight incubation, plates were washed three times with DPBS + 0.10% Tween-20, then incubated at room temperature with 50μL of biotinylated anti-porcine IFN-γ mAb P2C11 (1:1000; Mabtech). Plates were washed six times with DPBS + 0.10% Tween-20 then incubated with 100μL of 1:1000 streptavidin-alkaline phosphatase conjugate (1:1000 dilution; Mabtech). After 1hr incubation at room temperature, plates were washed six times with DPBS + 0.10% Tween-20 then developed by adding 100μL of BCIP/NBT (Plus) alkaline phosphatase substrate (Thermo Fisher). Development was stopped by washing several times with dH2O after spots appeared in the ConA positive control wells, the plates were air dried, and spots were enumerated on an automated ELISpot plate reader (Cellular Technology Ltd.). Results are expressed as number of spot-forming units (SFU) per 10^6^ PBMCs.

### 2.7 Influenza challenge and tissue collection

Six months after the initial immunization (D180) all swine were subjected to split intratracheal and intranasal inoculation (45) of a divergent IAV-S isolate under telazol (Zoetis), zolazepam (Zoetis), ketamine (Zoetis), and xylazine (Vet One) induced anesthesia. Swine were inoculated intratracheally with 2mL of 2.5x10^5^ TCID_50_/mL of A/swine/Wyoming/A01444562/2013 (sw/WY/13), and intranasally with 1mL 2.5x10^5^ TCID_50_/mL of virus per nostril for a total dose of 1x10^6^ TCID_50_ of virus. Swine were inoculated through both the intratracheal and intranasal route to ensure infection of both the upper and lower respiratory tract. Clinical disease was observed for the subsequent 4 days and scored by an experience veterinarian blinded to the treatment groups according to a previously established scoring system (46). Rectal temperatures were collected on 0-, 1-, 2-, 3-, and 4- days post infection and nasal swabs were collected on 1-, 3-, and 5-days post infection. Nasal swabs were placed in UniTranz-RT Universal Transport Medium (Puritan) then aliquoted and stored at -80°C. At 5dpi, all animals were euthanized with an overdose of sodium pentobarbital Fatal-Plus (Vortech), and lungs were removed for a bronchioalveolar lavage (BAL) wash and infectious virus quantification. One-centimeter-thick tissues were samples from the middle trachea, apical, middle, and caudal right lung was excised and stored in 10% neutral buffered formalin for H&E staining, histopathological analysis, and IHC against the conserved nucleoprotein (NP) viral antigen using a rabbit anti-Influenza A virus NP antibody (Cat. No. PA5-32242; Invitrogen). The formalin-fixed tissues were processed routinely for histologic examination after 72 hours fixation, sectioned at 4-5µm, and stained with hematoxylin and eosin and examined by an ACVP certified pathologist according to previously established scoring protocols (47). Tracheas were scored as 0, normal; 1, focal inflammation with cilia present; 2, diffuse inflammation and multi focal cilia loss; 3, widespread inflammation and cilia loss. Lung consolidation percentage was scored for apical, middle, and caudal lobes. Score were 0 normal, 1 <5%, 1.5 5-25%, 2.0 25-50%, 2.5 50-75%, 3.0 >75%. Scoring for bronchiolar necrosis, bronchiolar inflammation, septal inflammation, and perivascular cuffing was done on the lung sections with the highest consolidation score from each pig. The score 0-3 were used and reflected percentages of lung affected as described for other histologic lesions. IAV-S NP Immunohistochemistry distribution was scored on trachea and the apical lung lobe. Scores were 0 no stain, 1 trachea only, 2 trachea + bronchi, 3 trachea + bronchioles, and 4 trachea + bronchi + bronchioles.

### 2.8 RT-qPCR analysis in nasal swabs

Viral RNA was extracted from nasal swabs at 1-, 3-, and 5-dpi using the PureLink Viral RNA/DNA Extraction Kit according to manufacturer’s instructions (Invitrogen). Reverse-transcription qPCR was performed using the Luna Universal Prone One-Step RT-qPCR Kit (NEB) and analyzed on a QuantStudio 3 Real-Time PCR System (Applied Biosystems). The following cycling conditions were used: 55°C for 30 mins, 95°C for 2 mins, and 40 cycles of 95°C for 15 secs and 60°C for 30 secs. The universal primer-probe set for Influenza A Virus was used (BEI Resources, NR-15593, NR-15594, and NR-15595) and viral RNA quantification was calculated based on a standard curve created using RNA extracted from a known quantity of infectious virus of A/swine/Wyoming/A01444562/2013.

### 2.9 Tissue culture infectious dose

Presence of infectious virus in nasal swabs and bronchioalveolar lavage (BAL) was determined by titration on MDCK-Ln cells. Nasal swab and BAL samples were serially diluted in DMEM containing 5% FBS and 1% penicillin streptomycin then 2x10^5^ cells were added to virus dilutions and incubated for 24 hours. The next day, plates were washed twice with DPBS and DMEM containing 2μg/mL of TPCK-trypsin was added before incubating plates for 72 hours. After three days of incubation, 50μL of 0.5% chicken red blood cells were added and agglutination patterns were read after a 45-minute incubation at room temperature. All nasal swab samples and BAL samples were independently run with four technical replicates per sample.

### 2.10 Statistical analysis

All statistical analysis and data representation was carried out using GraphPad Prism 9. Data are expressed as the mean with standard error (SEM). HI titers, ELISA endpoint titers, T cell analysis, and TCID_50_ results were analyzed by one-way ANOVA with Tukey’s multiple comparisons. A *p* value <0.05 was considered statistically significant (*p < 0.05; **p < 0.01; ***p < 0.001; ****p < 0.0001).

## 3 Results

### 3.1 Ad-swH3-epigraph vaccination generates rapid and durable antibody responses

**Figure 2:**
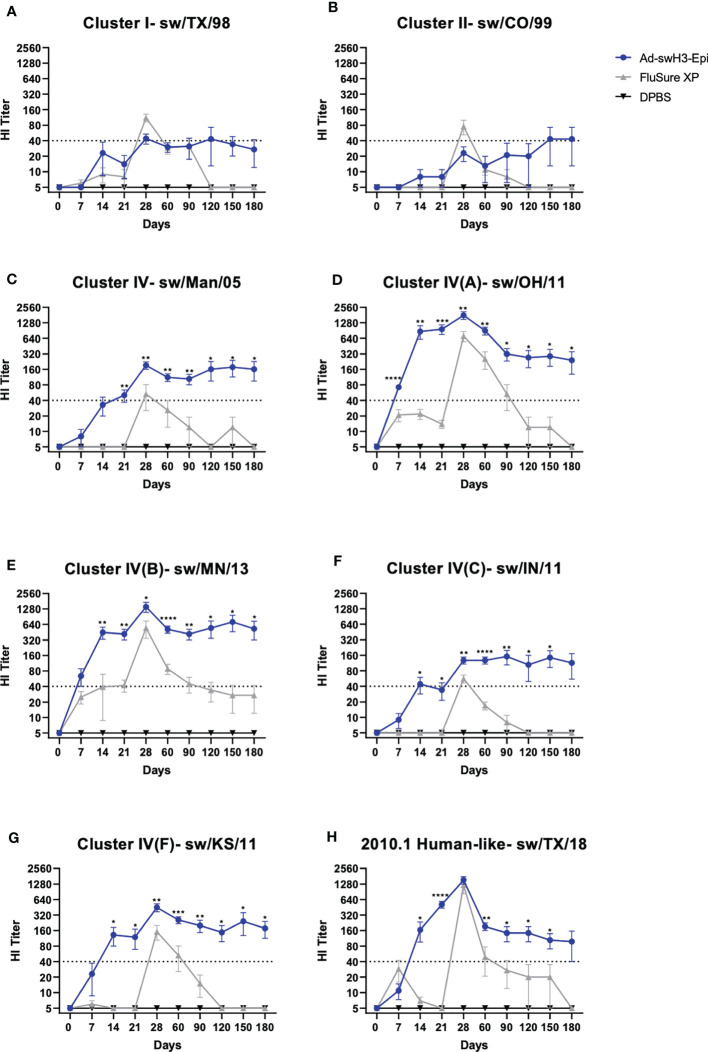
Vaccination with Ad-swH3-Epi rapidly induces durable antibody responses. Cross-reactive antibodies against Cluster I, Cluster II, and Cluster IV(A-F) and the human-like Cluster swine H3 strains were screened by hemagglutination inhibition (HI) assay. **(A)** Cluster I- sw/TX/98, **(B)** Cluster II- sw/CO/99, **(C)** Cluster IV- sw/Man/05, **(D)** Cluster IV(A)- sw/OH/11, **(E)** Cluster IV(B)- sw/MN/13, **(F)** Cluster IV(C)- sw/IN/11, **(G)** Cluster IV(F)- sw/KS/11, and **(H)** 2010.1 human-like Cluster- sw/TX/18. A protective titer of ≥1:40 is indicated as a dashed line on each graph. Data are represented as the mean ± SEM. (n=5; one-way ANOVA with Tukey’s multiple comparison; *p < 0.05, **p < 0.01, ***p < 0.005, ****p <0.0001).

To evaluate the onset and duration of antibody responses, groups of three-week-old cross-bred Yorkshire pigs were intramuscularly immunized with Ad-swH3-Epi and responses over time were compared to swine immunized with the commercial vaccine, FluSure XP, or DPBS as a negative control immunization group (**Figure 1A**). The breadth and duration of antibody responses were examined by hemagglutination inhibition (HI) assay against a panel of divergent IAV-S strains that represent Cluster I, Cluster II, Cluster IV (A-F), and the human-like Cluster of H3 IAV-S (**Figure 1B**). Immunization with FluSure XP and Ad-swH3-Epi induced moderate HI antibody responses against isolates representing Cluster I (**Figure 2A**) and Cluster II (**Figure 2B**) IAV-S. The responses to these cluster representatives exhibited similar kinetics of development and retraction over time with no statistically significant differences observed between FluSure XP and Ad-swH3-Epi immunized animals at any timepoints. Analysis of more recently circulating isolates from Cluster IV, Cluster IV subclusters A-F, and the 2010.1 cluster “human-like” IAV-S revealed that, while FluSure XP induced cross-reactive antibody responses after boost immunization, vaccination with Ad-swH3-Epi was able to rapidly elicit protective HI titers, represented by endpoint titers _ 40 (48–50), as early as two weeks after prime immunization (D14). HI titers induced by Ad-swH3-Epi vaccination were significantly higher than those observed in the FluSure XP immunized animals by 14 days post immunization (**Figures 2C-H**). Peak responses in the Ad-swH3-Epi immunized group were seen one week after the boost immunization (D28), and these responses were significantly higher than vaccination with FluSure XP against representative IAV-S isolates from Cluster IV (**Figure 2C**), Cluster IV(A) (**Figure 2D**), Cluster IV(B) (**Figure 2E**), Cluster IV(C) (**Figure 2F**), and Cluster IV(F) (**Figure 2G**). Notably, while protective responses _ 40 were observed after boosting in the FluSure XP immunized group, these responses rapidly dropped two months after the boost immunization (D90) to at or below the protective titer, and responses against sw/Man/05, sw/OH/11, sw/IN/11, sw/KS/11, and sw/TX/18 dropping to undetectable levels by the completion of the 6-month analysis. In comparison, pigs immunized with Ad-swH3-Epi had significantly more durable responses, with HI antibody levels gradually retracting over the course of 6 months (D180) and responses against sw/Man/05, sw/OH/11, sw/MN/13, sw/IN/11, sw/KS/11, and sw/TX/18 persisting at a level of _ 40 (**Figures 2C-H**) by the end of the 6-month study. These data suggest an exciting result because the average lifespan of a standard market pig is 6 to 7 months of age, and our vaccine demonstrates the ability to induce lasting protection against divergent IAV-S, potentially lasting the entire lifespan of market animals.

### 3.2 Ad-swH3-epigraph induces robust and durable T cell responses

**Figure 3:**
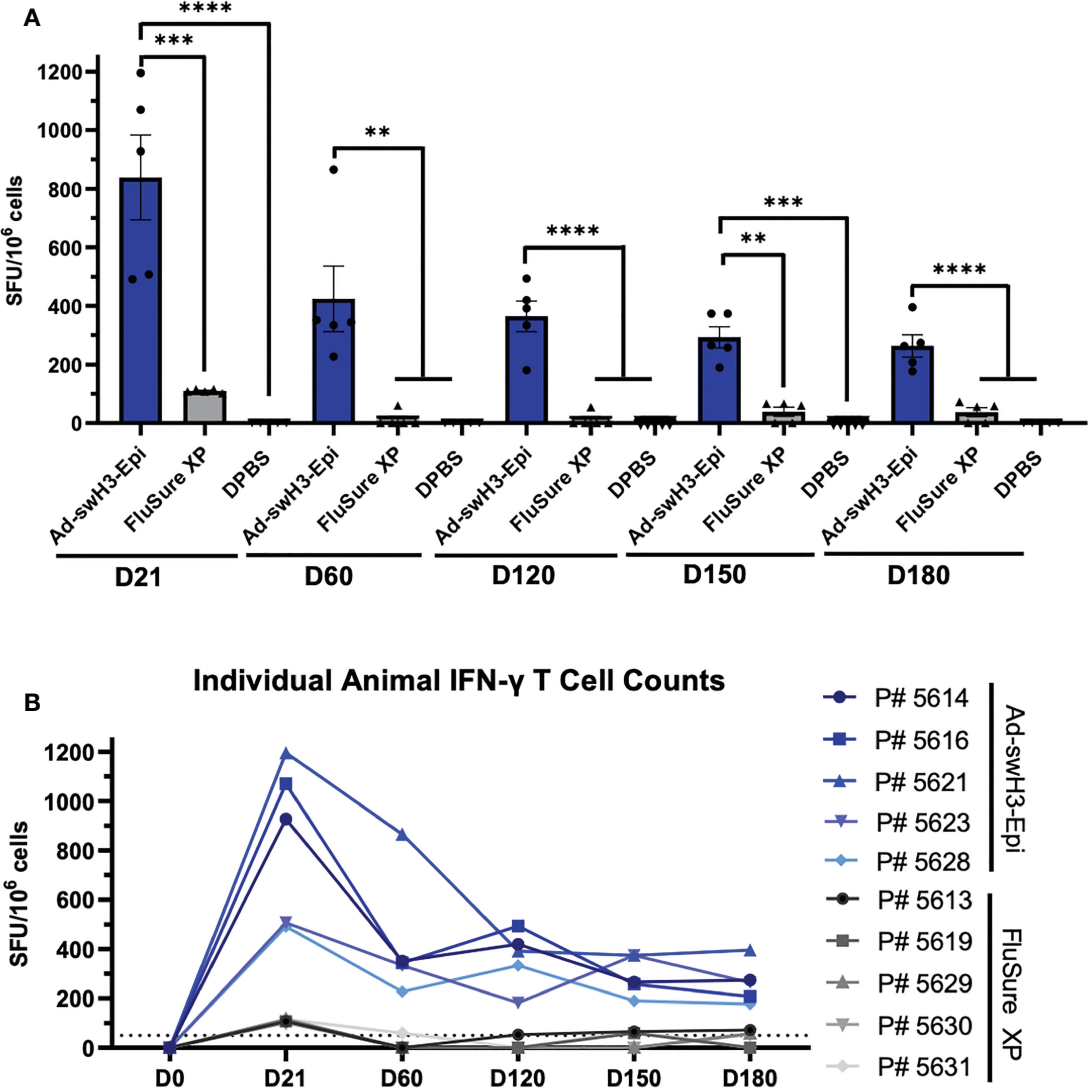
Vaccination with Ad-swH3-Epi elicits lasting T cell responses. PBMCs were isolated from peripheral blood at D0, D21, D60, D120, D150, and D180 and screened for antigen-specific T cells by IFN-γ ELISPOT. PBMCs were stimulated with an overlapping peptide array containing individual 17-mer peptides with 10 amino acid overlap spanning the entire length of A/swine/Ohio/11SW87/2011 HA protein. Peptide responses of ≥ 50 spot forming units (SFUs) per million PBMCs were considered positive. **(A)** Mean total T cell responses from D21 to D180 after vaccination with Ad-swH3-Epi, FluSure XP, or DPBS. Data are presented as the mean ± SEM (n=5 pigs/group; one-way ANOVA with Tukey’s multiple comparison; **p < 0.01, ***p < 0.005, ****p <0.0001). **(B)** T cell responses of individual animals in Ad-swH3-Epi (blue) and FluSure XP (grey) immunized groups analyzed over the study duration.

T cell responses have been shown to play an important role in viral clearance during influenza infection (51–53). In congruence with this, we evaluated the onset and duration of circulating T cell responses by IFN-γ ELISPOT against a Cluster IV(A) strain, A/swine/Ohio/11SW87/2011. An overlapping peptide array consisting of 17-mer peptides with 10 amino acid overlap was constructed, and responses were considered positive if greater than 50 spot forming units (SFU) were obtained per million cells analyzed (54). By D21, vaccination with Ad-swH3-Epi resulted in 7.6-fold higher levels of IFN-γ secreting T cells compared to FluSure XP immunized animals (mean 838 SFU/10^6^ cells compared to mean 110 SFU/10^6^ cells, respectively) (**Figure 3A**). While the circulating T cells of the pigs vaccinated with FluSure XP rapidly declined to undetectable or nearly undetectable levels by D60, circulating T cells induced by vaccination with Ad-swH3-Epi gradually retracted over time (D60 mean: 425 SFU/10^6^ cells; D120 mean: 364 SFU/10^6^ cells; D150 mean: 292 SFU/10^6^ cells; D180: 264 SFU/10^6^ cells) (**Figure 3A**), consistent with retraction of T cells in the absence of antigen stimulation over time. Individual analysis of each pig in the Ad-swH3-Epi and FluSure XP immunized groups showed peak responses at D21 for both groups, and all pigs in the Ad-swH3-Epi group maintained detectable T cell responses by D180 (**Figure 3B**). As expected, pigs in the DPBS immunization group did not produce any antigen-specific T cell responses (**Figure 3A**). Overall, we observed that vaccination with Ad-swH3-Epi induced strong circulating T cell responses that were significantly higher and more durable than those induced by vaccination with FluSure XP.

### 3.3 Ad-swH3-epigraph elicits faster and more robust class-switched IgG levels against a divergent human IAV hemagglutinin

**Figure 4:**
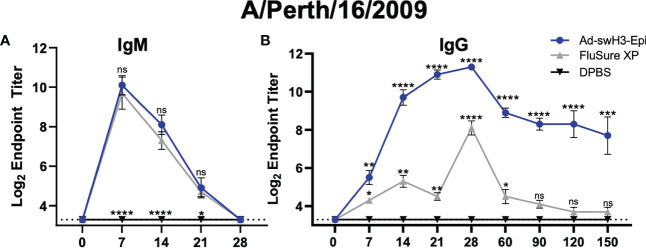
Vaccination with Ad-swH3-Epi allows for more robust class switching of IgM to IgG against a human H3 IAV strain. Sera from vaccinated pigs were isolated and used for antibody isotyping of IgM **(A)** and IgG **(B)** over time. Endpoint titers are represented as log_2_ of the reciprocal of the highest sera dilution that were positive against A/Perth/16/2009 recombinant protein. Samples were considered positive if the OD value was three standard deviations above the mean OD values measured from negative control immunized animals. Data are represented as the mean ± SEM (n=5 pigs/group; one-way ANOVA with Tukey’s multiple comparison; *p<0.05, **p < 0.01, ***p < 0.005, ****p <0.0001). All statistical significance shown in the Ad-swH3-Epi group are compared to FluSure XP immunized pigs. All statistical significance shown in the FluSure XP group are compared the DPBS immunized pigs. ns, not significant.

We next assessed the kinetics of antibody class switching to detect antigen-specific IgM and IgG antibodies in the peripheral blood. Antibodies from Ad-swH3-Epi, FluSure XP, and DPBS immunized pigs were screened by ELISA against a divergent human H3 IAV recombinant HA protein, A/Perth/16/2009. We observed a similar onset and duration of IgM antibody responses mounted between the Ad-swH3-Epi and FluSure XP immunized animals with no statistically significant differences in IgM antibody levels at D7, D14, D21, or D28 (**Figure 4A**), showing that the rate of IgM antibody development was not different between these two vaccination platforms. DPBS immunized animals did not develop any IgM antibody responses, as expected (**Figure 4A**). However, when we assessed the levels of class-switched antigen-specific IgG antibody levels, we observed that pigs vaccinated with Ad-swH3-Epi developed antigen-specific IgG antibodies sooner than those observed in the FluSure XP immunized pigs (**Figure 4B**). Similarly, these responses were elicited at significantly higher levels than those induced after vaccination with FluSure XP, which retracted by D90 and were not significantly higher than unimmunized pigs from D90 to D150. The similar kinetics of IgM levels between Ad-swH3-Epi and FluSure XP immunized pigs but differences in mounted of antigen-specific IgG levels is likely due to a stronger T cell development in Ad-swH3-Epi vaccinated pigs (**Figure 3**), which are crucial in triggering class-switch recombination and strong plasma cell development and subsequently IgG antibody development (55, 56). These results suggest that development of robust and broadly reactive IgG antibody responses can be achieved by vaccination with Ad-swH3-Epi, and that these responses are significantly higher than those observed with the whole inactivated virus vaccine, FluSure XP.

### 3.4 Ad-swH3-epigraph vaccination reduces viral shedding after heterologous challenge

**Figure 5:**
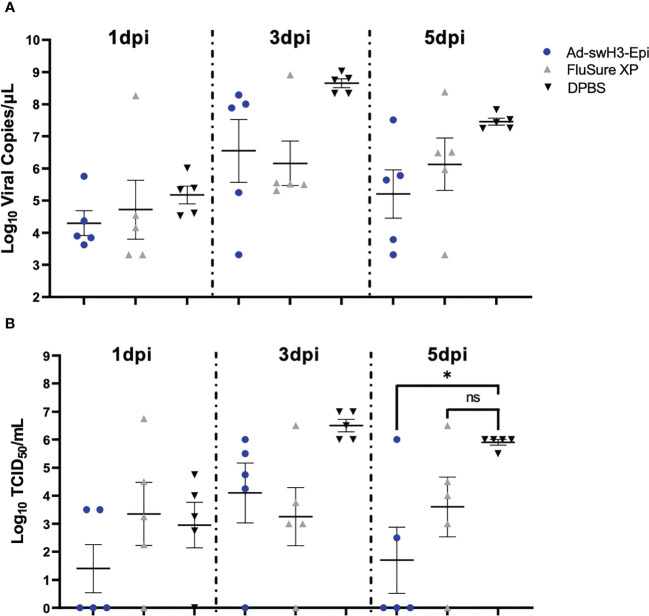
Ad-swH3-Epi reduces viral shedding at 5 days post infection. Swine were challenged with 10^6^ TCID_50_ of A/swine/Wyoming/A01444562/2013 by split intratracheal and intranasal inoculation. Nasal swabs were collected at 1-, 3-, and 5-days post infection and amount of viral RNA was quantified by RT-qPCR **(A)** and levels of infectious virus were measured by TCID_50_ **(B)**. Data are presented as the mean ± SEM (n=5 pigs/group; one-way ANOVA with Tukey’s multiple comparison; *p<0.05). ns, not significant.

We next wanted to assess the ability of Ad-swH3-Epi and FluSure XP to provide protection against heterologous challenge six months after the initial vaccination (D180). This objective is particularly important because the average time to grow and procure a market pig is typically 6-7 months (57) and, given that IAV-S affects swine at all stages of pork production, providing protection against challenge for six months could greatly impact pork production outcomes and limit the spread of IAV-S among pigs during production. All pigs were challenged with 10^6^ TCID_50_ of a Cluster IV(A) IAV-S isolate, A/swine/Wyoming/A01444562/2013 (**Figure 1A**), by split intratracheal and intranasal inoculation (45) under anesthesia. This strain was chosen based on epidemiological analysis of recently circulating strains within the United States (9) and may represent isolates that swine could interface in the field. Clinical disease was scored by an experienced veterinarian blinded to the treatment groups and rectal temperatures were collected daily. No significant differences in clinical disease or change in body temperature was observed between the three vaccination groups (**Supplementary Figure 1A, B**), which is likely due to the advanced age of the pigs at the time of challenge (45). Nasal swabs were collected at 1 day post infection (dpi), 3dpi, and 5dpi. Presence of viral RNA was assessed by RT-qPCR and quantification of infectious virus was enumerated by TCID_50_. At 1dpi, 3dpi, and 5dpi similar levels of viral RNA was detected among all groups, with no statistically significant differences observed at any timepoint (**Figure 5A**). When analyzing the presence of infectious virus by TCID_50_ a trend of lower levels of infectious virus were seen at 1dpi in Ad-swH3-Epi group compared to FluSure XP and DPBS immunized animals, though this did not reach statistical significance (**Figure 5B**). Similar levels of infectious virus were present at 3dpi, with no statistically significant differences observed between the three immunization groups. Importantly, at 5dpi pigs vaccinated with Ad-swH3-Epi showed to have significantly lower presence of infectious virus in nasal secretions compared to DPBS immunized pigs, while FluSure XP immunized pigs had similar levels of infectious virus as DPBS immunized pigs (**Figure 5B**). This result suggests that vaccination with Ad-swH3-Epi can resolve viral shedding earlier and lower the risk of transmission of infectious virus through nasal secretions.

### 3.5 Ad-swH3-epigraph reduces microscopic lesion development, lowers presence of infectious virus and viral antigen in the lungs, and provides strong reactivation of circulating antigen-specific T cells after challenge

**Figure 6:**
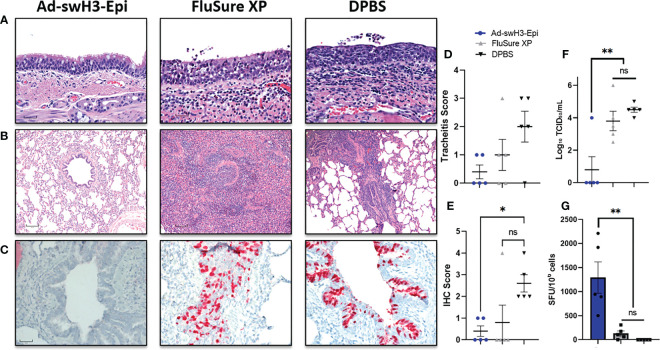
Ad-swH3-Epi provides protection against challenge with a divergent IAV-S. Five days after challenge lungs and tracheas were removed for histopathological analysis. H&E staining of representative trachea samples are shown **(A)** and representative bronchioles from lungs are shown in **(B)**. Immunohistochemistry of bronchioles against the conserved NP viral protein **(C)**. Tracheas shown in **(A)** were scored for tracheitis by a board-certified pathologist blinded to the treatment groups **(D)**. IHC distribution was recorded; higher scores correlate with deeper pulmonary infection **(E)**. Levels of infectious virus present in the bronchioalveolar lavage was enumerated by tissue culture infectious dose (TCID_50_) **(F)** and recall T cell responses were analyzed by IFN-γ ELISPOT **(G)**. Scale bars in **(A–C)** are 30μM, 120μM, and 60μM, respectively. Data are presented as the mean ± SEM (n=5; one-way ANOVA with Tukey’s multiple comparison; *p < 0.05 and **p < 0.01). ns, not significant.

Lastly, all pigs were humanely bled and euthanized at 5 days post infection for histopathological analysis of lung and tracheal tissues, quantification of viral antigen and infectious virus in the lungs, and evaluation of recall T cell responses. Gross lesions were observed on the lungs of one FluSure XP immunized pig (**Supplementary Figure 2**) and were not present in any other vaccine group. Histopathological analysis of trachea samples (**Figure 6A**) showed that pigs vaccinated with Ad-swH3-Epi displayed healthy submucosal tissues with minimal infiltration of inflammatory cells and respiratory epithelial cells that were columnar and ciliated. In contrast, vaccination with FluSure XP resulted in inflammatory cell infiltrates into the submucosa and mucosal epithelium with rounded and cuboidal respiratory epithelial cells and reduction in cilia at the epithelial surface. DPBS immunized pigs showed significant inflammatory cell infiltrates coupled with disrupted and thinned surface epithelium and complete loss of cilia. This resulted in a trend of decreased tracheitis scoring in the Ad-swH3-Epi group compared to FluSure XP and DPBS immunized pigs (**Figure 6D**). Further analysis of sectioned bronchioles revealed that vaccination with Ad-swH3-Epi was able to protect against bronchiolar necrosis, inflammation, and perivascular cuffing (**Figure 6B**). In comparison, FluSure XP and DPBS immunization demonstrated significant suppurative bronchitis and inflammatory cell infiltration into the bronchiole (**Figure 6B**). Immunohistochemical (IHC) analysis against the conserved influenza nucleoprotein (NP) antigen showed many viral infected cells within the epithelium of bronchi and bronchioles of pigs immunized with FluSure XP or DPBS (**Figure 6C**) leading to a higher IHC score in these groups (**Figure 6E**). A bronchioalveolar lavage (BAL) was performed during necropsy to evaluated levels of infectious virus present in the lungs by TCID_50_. High titers of infectious virus were present in the BAL obtained from pigs immunized with FluSure XP and DPBS (FluSure XP mean: 10^3.8^ TCID_50_/mL; DPBS mean: 10^4.5^ TCID_50_/mL) (**Figure 6F**). In contrast, only one pig in the Ad-swH3-Epi immunization group had detectable levels of infectious virus present in the BAL (Ad-swH3-Epi mean: 10^0.8^ TCID_50_/mL) while all other pigs had cleared the virus from the lungs by 5 days post infection (**Figure 6F**). Finally, we evaluated the recall T cell responses elicited after challenge by assessing levels of antigen-specific T cells present in the peripheral blood at 5dpi by IFN-γ ELISPOT against the same overlapping peptide array previously used to assess the development and duration of T cell responses after vaccination (**Figure 3**). We observed a strong reactivation of circulating antigen-specific T cell responses (**Figure 6G**) that were higher than those observed at D21 after vaccination (**Figure 3A**) (D21 mean: 838 SFU/10^6^ and 5dpi mean: 1293 SFU/10^6^ cells). This demonstrates that antigen encounter after challenge was able to robustly reactivate memory T cells and likely played a crucial role in the clearance of viral infected cells after challenge. Collectively, we have demonstrated that the immune responses elicited after immunization with Ad-swH3-Epi can protect against challenge with a heterologous IAV-S isolate 6 months after vaccination and this protection is significantly better than the protection observed after immunization with WIV vaccine, FluSure XP.

## 4 Discussion

In this study, we characterized the onset and duration of immune responses elicited after vaccination with Ad-swH3-Epigraph and compared these responses to a commonly used WIV vaccine, FluSure XP. An ideal vaccine for use against IAV-S in pigs would elicit lifelong responses that provide protection against a broad range of antigenically distinct viruses. However, current methods of vaccination fail to provide durable and broadly protective responses and can induce vaccine-associated enhanced respiratory disease (VAERD) after heterologous challenge. Here, we demonstrate that vaccination with Ad-swH3-Epi rapidly induced robust and durable antibody responses against recently circulating IAV-S isolates that remained above protective levels (_ 40) for 6 months after vaccination. In comparison, FluSure XP did not induce protective titers until after boost immunization, corroborating previously identified results (41). Further, antibody levels in the FluSure XP immunized pigs quickly diminished to below protective titers 30-60 days after boost vaccination, indicating that this vaccine platform may induce a short duration of antibody-mediated protection. The duration of these responses has significant clinical relevance, as the average lifespan of a standard market pig in the pork industry is 6-7 months of age, and IAV-S affects swine at all stages of pork production. Interestingly, we also saw modest antibody responses against the representative Clade I and Clade II IAV-S isolates, sw/TX/98 and sw/CO/99, in the Ad-swH3-Epi immunization group. Indeed, we have previously observed lower responses to these clades (41), which is likely due to the limited representation of these strains in the Epigraph immunogen design.

Importantly, we also observed robust and durable antigen-specific T cell development after vaccination with Ad-swH3-Epi. While FluSure XP immunized pigs developed modest T cell responses that quickly retracted by D60, Ad-swH3-Epi was able to induce lasting responses that were detectable 6 months after vaccination. Given that T cells play a pivotal role in the clearance of viral infected cells, B-cell activation, and class-switching during affinity maturation, we hypothesize that the strong induction of T cell responses likely played an important role in the development of class-switched antigen-specific IgG antibody levels as well as the viral clearance observed after challenge. While we observed similar kinetics of IgM development between the two immunization groups, we also observed reduced levels of class-switched IgG antibodies with a delayed onset in the FluSure XP immunized pigs compared to Ad-swH3-Epi immunized animals. This observation, coupled with the kinetics of robust T cell activation, suggests that the enhanced levels and duration of antibody responses seen in the Ad-swH3-Epi group were due to a balanced induction of both B and T cell responses.

Recent epidemiological analysis of U.S. swine herds indicates high circulation of Cluster IV(A) H3 IAV-S (9). In accordance with this, we chose to challenge the pigs with a high dose of divergent IAV-S isolate, A/swine/Wyoming/A01444562/2013, 6 months after the initial vaccination. We hypothesized this challenge study design closely recapitulates relevant field conditions. Further, this isolate was not screened for antibody or T cell responses prior to challenge to prevent bias in choosing the challenge strain. Analysis of nasal swabs indicated that vaccination with Ad-swH3-Epi was able to reduce the duration of viral shedding, as 3/5 pigs had completely abrogated levels of infectious virus present in the nose by 5dpi. This is important because reduction of infectious virus in the nose can reduce transmission and spread of IAV-S between pigs within a herd and lower the potential of zoonotic transmission of IAV-S into humans at swine-to-human interfaces (58). Further analysis of the lungs and trachea indicated that the durable immune responses elicited by Ad-swH3-Epi immunization was able rapidly clear infected cells, as pigs exhibited reduce levels of tracheitis, healthy bronchioles, and reduced viral antigen detected by IHC. This was coupled with significant reduction of infectious virus in the lungs by 5dpi. We further characterized that influenza challenge was able to activate robust recall T cell responses in the Ad-swH3-Epi immunized animals, corroborating the crucial role of T cells during protection against IAV-S in swine. Here, we observed that Ad-swH3-Epi induced broad and durable protection that was significantly superior to a commonly used WIV vaccine, FluSure XP.

Recent advances in gene delivery by viral vectors have paved the way for improved safety and immunogenicity profiles of viral vectors. In light of the recent COVID-19 pandemic, two adenoviral-vectored vaccines have recently made it to the market for use in humans (59–61). Utility of a species C human adenovirus type 5 (Ad5) viral vector has been well described in both humans and swine against a variety of infectious diseases. Notably, the tissue tropism of Ad5 in swine has been identified as lung epithelial cells (62, 63) and pulmonary intravascular macrophage cells (64, 65), which is an optimal location to elicit both antibody and cellular-mediated immune responses against a respiratory pathogen such as influenza. Despite the substantial advances in adenoviral-vectored vaccine development, there is still a concern of dampened responses due to preexisting immunity to the delivered vector after boosting with a homologous adenovirus serotype. By utilizing a human adenovirus as our vaccine vector, we minimize the risk of swine having previously been exposed to the vector through natural infection mechanisms. Further, while we observed peak antibody responses after boosting, it is possible that boosting with a heterologous adenovirus serotype would have elicited higher levels of antibody responses and T cell responses. Additional studies investigating heterologous adenovirus prime-boosting strategies in swine are needed to fully elucidate the potential to induce stronger responses than those observed in this study.

The results obtained in this study have significant impact to the field of vaccine development and details the kinetics of immune responses elicited after vaccination with WIV vaccines compared to viral-vectored vaccination strategies. Notably, standard methods of vaccine efficacy testing typically analyze and report short durations of immune responses and perform *in vivo* challenges shortly after vaccination during peak responses. Here, we chose to sequentially analyze immune responses in a longitudinal study to mitigate possible biases in vaccine efficacy testing. However, completing a longitudinal study in swine can have several limitations. One such limitation is the rapid growth of domestic swine, which requires advanced containment facilities, enhanced biocontainment practices after challenge, and specially trained personnel for animal handling. An additional limitation is that older swine have been characterized to be less susceptible to IAV-S infection compared to younger pigs (45). To overcome these challenges, we chose to use a high dose of IAV-S inoculation to ensure adequate infection in our *in vivo* infection model and were able to collect infectious virus from DPBS immunized pigs at 5dpi, indicating that these older pigs were susceptible to IAV-S infection. A final limitation of this study was that we were only able to perform a challenge using one virus. Though our challenge strain was chosen to represent recently circulating strains, reverse-zoonotic transmission of IAV from humans to swine has established a stable cluster of IAV-S, known as the 2010.1 cluster, that has recently emerged as an endemic clade within U.S. swine herds (66). While we were unable to perform an additional challenge in this longitudinal study, we hypothesize that we would see similar protection against this clade, as we observed robust and durable HI antibody responses to the representative 2010.1 strain, sw/TX/18. However, further challenge studies are required to confirm this hypothesis.

Here, we characterized the onset and duration of immunity elicited by vaccination with an adenoviral vectored Epigraph vaccine and compared these responses to a commonly used WIV vaccine, FluSure XP. We observed that vaccination with Ad-swH3-Epi induced durable and protective levels of antibody and T cell responses that remained detectable for 6 months, and a faster evolution of class-switched IgG antibody responses compared to FluSure XP. We further identified that these responses lead to significantly reduced viral shedding by 5dpi, enhanced viral clearance from the lungs, and prevented the development of lesions in the trachea and lungs after challenge 6 months post-vaccination. The results obtained in this study can enhance to our understanding of immune responses elicited after vaccination in swine and contribute to the development of a universal swine influenza virus vaccine.

## Data availability statement

The original contributions presented in the study are included in the article/**Supplementary Material**, Further inquiries can be directed to the corresponding author.

## Ethics statement

The animal study was reviewed and approved by University of Nebraska - Lincoln, Institute of Animal Care and Use Committee (Protocol: 2167).

## Author contributions

EP-T, MP, NJ, and CW provided technical expertise and performed sampling, animal husbandry and management, performed assays and data collection. EP-T, MP, and CW performed immune assays and curated samples collected during the studies. EP-T, DS and HV performed the swine challenge experiments, collected data, and analyzed data. EP-T collected all data and performed extensive analyses and statistics. EP-T, DS and HV interpreted the histology and EAW designed, managed, supervised and procured funding for the research study. EP-T and MP wrote the manuscript. All authors contributed to the article and approved the submitted version.

## Supplementary material

Supplementary Figure 1 Daily rectal temperatures and daily clinical disease scoring after infection. **(A)** Daily rectal temperatures were collected after infection. A dashed line at 38.4°C indicates normal body temperatures of swine. A dashed line at 39.5°C indicates pyrexia in swine. **(B)** Daily clinical disease scoring was done by an experienced vet blinded to the treatment groups. A previously established scoring scale from 1-5 was used.

Supplementary Figure 2 Gross lesion analysis of lungs 5 days after challenge. Lungs were removed at 5dpi and representative lungs from each vaccination group are shown. Lungs were observed for purple-red consolidation on the lobes and minor consolidation was noted on one animal in the FluSure XP immunization group (yellow arrows).
