# Supplementary figures and images for: Adenoviral-vectored epigraph vaccine elicits robust, durable, and protective immunity against H3 influenza A virus in swine
Source: Front Immunol. 2023 May 15;14:1143451. doi: 10.3389/fimmu.2023.1143451 (PMC10225514; doi:10.3389/fimmu.2023.1143451)

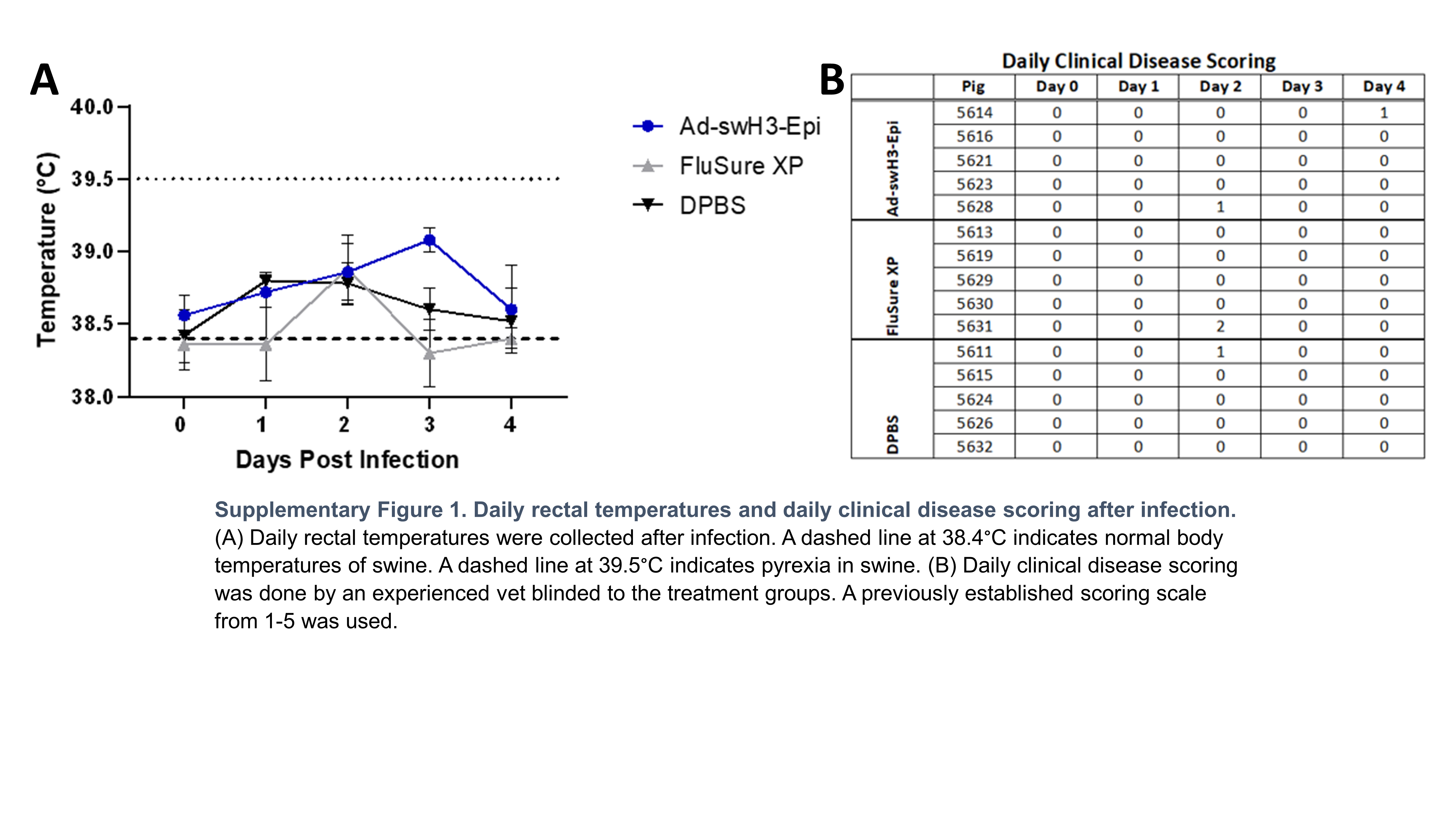

Supplement: Supplementary Figure 1 — Daily rectal temperatures and daily clinical disease scoring after infection. (A) Daily rectal temperatures were collected after infection. A dashed line at 38.4°C indicates normal body temperatures of swine. A dashed line at 39.5°C indicates pyrexia in swine. (B) Daily clinical disease scoring was done by an experienced vet blinded to the treatment groups. A previously established scoring scale from 1-5 was used. [file Image_1.tif]

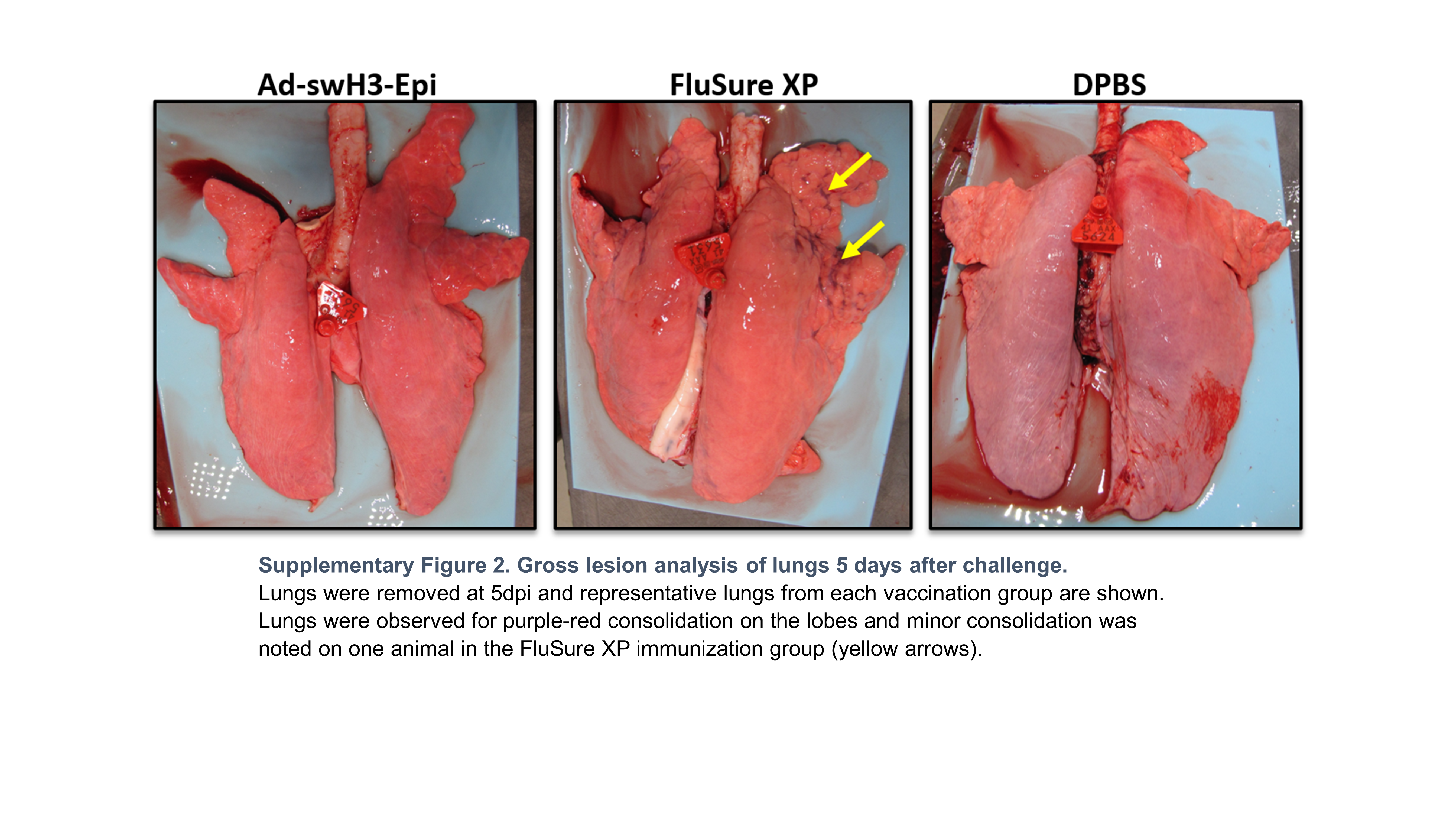

Supplement: Supplementary Figure 2 — Gross lesion analysis of lungs 5 days after challenge. Lungs were removed at 5dpi and representative lungs from each vaccination group are shown. Lungs were observed for purple-red consolidation on the lobes and minor consolidation was noted on one animal in the FluSure XP immunization group (yellow arrows). [file Image_2.tif]
